# Consumers' Perception of Generic Medicines and Evaluation of In Vitro Quality Control Parameters of Locally Manufactured Paracetamol Tablets in Asmara, Eritrea: A Cross-Sectional Study

**DOI:** 10.1155/2021/6642826

**Published:** 2021-03-26

**Authors:** Idris Mohammed Idris, Diyae Nesredin Hassan, Hanan Abdelkadir Hassen, Rahwa Zerabruk Araya, Dawit G. Weldemariam

**Affiliations:** ^1^Department of Anesthesia, Eritrean Air Force Military Hospital, Asmara, Eritrea; ^2^Department of Medical Sciences, Orotta College of Medicine and Health Sciences, Asmara, Eritrea; ^3^Department of Pharmacy, Hazhaz Zonal Referral Hospital, Asmara, Eritrea

## Abstract

Generic medicines are clinically equivalent and can be used interchangeably for their intended use. Globally, the usage of generic medicines is highly recommended because of their affordability and accessibility. However, consumers hold a negative perception and attitude of using generic medicine as they consider it poor and having inferior quality compared to branded medicines. This study was conducted to assess the consumers' general view of generic medicines and in vitro evaluation of a locally produced generic medicine, paracetamol. An analytical and cross-sectional study was conducted in three selected hospitals, and in vitro quality control evaluation was done in National Drug Quality Control Laboratory between October 26 and November 21, 2017, in Asmara, Eritrea. A systematic random sampling design was employed, and the data was collected using a questionnaire and a check-list for recording the quality control parameters of paracetamol tablets. A total of 403 respondents were included in the study. The majority of the study participants were females (61.8%). Generally, about half (49.1%) of the respondents choose locally manufactured paracetamol over the imported ones. More than half (68.5%) of the respondents did not believe expensive medicines are of better quality. The main reason consumers prefer the local paracetamol (Azemol) tablet to the imported one was due to their good experience (62.1%). About three-fourths (78.1%) of the consumers also believed that medicines manufactured abroad confer higher quality. At the multivariate level, having educational backgrounds such as elementary (AOR = 4.19, 95% CI: 1.251, 14.035) and junior (AOR = 2.4, 95% CI: 1.146, 5.028) was associated with preferability to local paracetamol as a pain killer over the brand ones. The in vitro test of the local paracetamol met the standard specification for the identification test, weight variation test, pharmacopeial test, friability test, disintegration test, and dissolution test. In conclusion, the majority of the consumers considered local paracetamol as having an inferior quality when compared with brand paracetamol. However, the reality revealed that the local paracetamol was of the same quality as the brand ones. To facilitate widespread use of generic medicines, healthcare professionals should educate consumers on the advantages of these medicines.

## 1. Introduction

Generic medicines (GMs) provide similar and a cost-effective alternative to branded medicines. They consist of equal active ingredients, the same strength as branded medicines [1, 2]. According to the World Health Organization (WHO), GM is defined as “a pharmaceutical product, usually intended to be interchangeable with an innovator product that is manufactured without a license from the innovator company and marketed after the expiry date of the patent or other exclusive rights” [1, 3]. Generic medicines are required to be equivalent to the originator product in terms of their strength, safety, efficacy, quality, pharmaceutical dosage form, and route of administration, and they can differ in excipients, color, and shape [1, 4, 5]. Thus, GMs need to comply with bioequivalence testing, the fundamental regulatory requirement, prior to its approval [6]. The United States Food and Drug Administration (US-FDA) defines bioequivalence as the absence of a significant difference in the rate and extent to which the active ingredient or active moiety in pharmaceutical equivalents or pharmaceutical alternatives becomes available at the site of drug action when administered at the same molar dose under similar conditions in an appropriately designed study [7].

As GMs are 20-90% cheaper than innovative medicines, they often reduce government's pharmaceutical health care expenditure and provide saving to a patient [8, 9]. Governments and third-party payers promoted the use of GMs to limit the increasing health care costs without compromising health care quality [5]. In 2011, the use of US-FDA-approved generic medicines saved 158 billion dollars, and in the United States, the use of GM saved over 1 trillion dollars between 2003 and 2014 [1, 10]. Therefore, a significant health care savings can be achieved if countries employ generic policies [11]. It is important to allow generic substitution and generic prescription in the health care system to contain the increasing cost of medicines [5]. Many countries, like the UK, US, and France, advocate the use of GMs to limit spending and improve affordability [12]. Although many countries have introduced generic prescriptions and generic substitutions, the use of GMs is limited due to inadequate knowledge and negative beliefs among consumers and health care professionals [6, 7]. Consumers should be knowledgeable about generic medicine and branded medicines as it is essential for acceptance of generic substitution to contain their expenditures [13].

In Eritrea, there are some commonly known branded medicines to the public that include but not limited to Panadol (paracetamol), Advil (ibuprofen), and Bactrim (cotrimoxazole). The paracetamol tablet is approved by the Eritrean regulatory authority and is produced by AZEL Pharmaceutical Share Company located in Keren, Eritrea. The pharmaceutical company is licensed to manufacture paracetamol (Azemol) in different dosage forms and strengths. It is generally known that paracetamol tablet is one of the most consumed over the counter (OTC) medicines in its generic and brand form. As it was shown in a study done to assess consumer's self-medication practice of OTC medicines, OTC analgesics were one of the most used medicines in Asmara, Eritrea [14]. OTC analgesics manufactured in abroad are believed to confer superior quality than their generic counterpart.

To the best of our knowledge, there were no previous studies conducted to assess consumers' view on generic medicine usage in Eritrea. Thus, the aim of the study was to assess the consumer's view and reality on generic medicines, specifically paracetamol tablet, manufactured locally in the outpatient departments of three selected hospitals in Asmara, Eritrea.

## 2. Methods

### 2.1. Study Design and Setting

An analytical and cross-sectional study was conducted to assess consumers' perception on generic medicines using a questionnaire in three selected hospitals: Halibet National Referral hospital, Orotta National Referral hospital, and Sembel hospital. Halibet and Orotta Hospitals are national referral hospitals, whereas Sembel hospital is a privately owned hospital in Asmara, Eritrea.

The in vitro evaluation was conducted in the National Quality Control Laboratory, National Medicines and Food Administration (NMFA), located in Asmara, the capital city of Eritrea. The data was collected between October 26 and November 21, 2017.

### 2.2. Study Population

All ambulatory patients above 18 years old, who visited the three selected hospitals, were the study population.

### 2.3. Sampling Design and Sample Size Determination

The sample size was calculated using this formula: *n* ≥ *Z*^2^*p*(1 − *p*)/*d*^2.^

The total sample size was calculated based on the following assumptions: 50% level of precision, absolute precision of (*p*) 0.05, *Z* statistics for 95% level of confidence (*Z* = 1.96), margin of error (*d*) of 0.05, and 10% nonresponse rate. Considering the above assumptions, the sample size was found to be at least 412.

The participants were selected using a systematic random sampling. First, the number of participants to be interviewed was proportionally allocated to the three hospitals; then, the average number of patients per OPD department per day was estimated. A random number was selected, and then every *n*^th^ number was taken from the queue.

For the in vitro test of paracetamol, two batches of local paracetamol tablets with each batch constituted of 100 tablets (10 strips) were used for the quality control test. These samples were purchased from the community chain pharmacies.

### 2.4. Data Collection and Variable Measurements

A self-developed and interviewer-based questionnaire was used to collect the data. The face and content validity of the questionnaire were assessed by various experts in the fields of pharmacy, public health, and medicine. Prior to initiation of the study, the questionnaire was pretested in 20 patients in Hazhaz hospital. The questionnaire consists of two sections. Section A encompassed 10 questions that intended to record information regarding the sociodemographic characteristics of the participants such as age, gender, nationality, place of residence, religion, ethnicity, marital status, and occupation. Section B consisted of 14 questions tried to explore the consumers understanding of the quality of medicine, whether this understanding affects their overall perception.

During the data collection process, the data collectors were showing the local paracetamol (Azemol) to the participants to ensure the accuracy of the collected data. The participation of patients was strictly voluntary, and informed consent was obtained before initiation. The patients were approached when they were waiting in the queue, before they were called by their doctors, and were briefed about the objectives of the study.

Two batches of 10 strips of paracetamol, each containing 10 tablets, were collected from community pharmacies for the in vitro quality analysis. The name, batch number, name of the manufacturer, manufacturing, and expiration date were checked before collection. In this study, the two batches were coded as PARA-1 and PARA-2.

The in vitro quality analysis was performed to determine the overall paracetamol quality. It included tests for identity, disintegration, assay, dissolution, stability, sterility, impurity, bioavailability, and bioequivalence as per the monograph of British pharmacopeia (2009). This study was focused on assessing the weight variation test, identification, pharmacopeial assay, friability, disintegration, and dissolution test.

### 2.5. In Vitro Quality Analysis Materials and Procedure

Laboratory instruments used in the in vitro quality analysis included analytical weighing balance, friability test apparatus, dissolution test apparatus, tablet hardness tester, and UV-spectrophotometer. Moreover, the reagents used were sodium hydroxide, potassium dichromate, and hydrochloric acid.

### 2.6. Weight Variation Test

This test was aimed to know the content uniformity of tablets. Twenty tablets were taken from each batch and weighed individually using an analytical balance (sartorus cp 2250). Then, the average weight for each batch was calculated. The acceptable limit for the deviation of weight for tablets having an average weight of 250 mg or more should not exceed +5% [15]. For all tablet brands, the following mathematical equation was used for weight variation:

(1) $$\begin{array}{c} \%\text{of weight variation}=\frac{\text{Highest weight}-\text{Average weight}}{\text{Average weight}}\times 100. \end{array}$$

### 2.7. Identification Test

Three tablets from each batch were weighed, and the average weight which contains 0.5 g of paracetamol was calculated and powdered.

The quantity of powdered paracetamol tablets containing 0.5 g of paracetamol was extracted with 20 ml of acetone. The extract was filtered, and the filtrate was evaporated and dried at 105 degrees Celsius. The residue had to comply with the following test: 0.1 g of the dried residue was boiled with 1 ml of hydrochloric acid for 3 minutes, and 10 ml of water was added and was then left to cool. After no precipitate production was observed, 0.05 ml of 0.0167 M potassium dichromate was added to see the appearance of violet color which does not turn red.

### 2.8. Pharmacopeial Assay Test

Twenty tablets were weighed and powdered. A quantity of the powder containing 0.15 g of paracetamol was added to 50 ml of 0.1 M sodium hydroxide. It was diluted with 100 ml of water and shaken for 15 minutes using a sonicator. Sufficient water was added to produce 200 ml, and the resulting solution was then mixed and filtered. 10 ml of the filtrate was diluted to 100 ml with the use of water. 10 ml of the resulting solution was added to 10 ml of 0.1 M sodium hydroxide and was diluted to 100 ml with water. The absorbance of the resulting solution was measured at the maximum at 257 nm.

### 2.9. Friability Test

This method is also called as the attrition-resistance method and testing the ability of a tablet to resist mechanical fracture during handling and manufacturing [16, 17].

Ten tablets from each batch were initially weighed and transferred into a friability test apparatus. The apparatus was operated at 25 rpm for 4 minutes (up to 100 revolutions). The tablets were weighed again, and the percent (%) friability was then calculated by using a formula. Generally, the considerable range of weight loss of conventional compressed tablet is less than 1% [16, 17].

(2) $$\begin{array}{c} \%\text{of friabiity}=\frac{\text{ Weight before test}-\text{Weight after test}}{\text{Weight after test}}. \end{array}$$

### 2.10. Disintegration Test

Dissolution tests were performed to determine the rate and amount of active medicines going into solution in a specified medium. It was performed in accordance of the specifications of the British Pharmacopoeia drug monographs (2009) using Apparatus II (paddle apparatus). The amount of drug going into solution at the respective times was measured using UV-spectrophotometer and compared to the specifications of the British Pharmacopoeia (2009).

### 2.11. Dissolution Test

It is an important test which measures the amount of drug release from the solid dosage form and predicts the bioavailability of the drug [16].

900 ml of phosphate buffer pH 5.8 was used as the medium, and the dissolution tester (paddle apparatus) was rotated at 50 revolutions per minute. A sample of 20 ml of the medium was withdrawn periodically as per the official monograph and filtered. The filtrate was diluted with 0.1 M sodium hydroxide through a serial of dilutions to give a solution expected to contain a concentration of about 0.00075% w/v of paracetamol.

The absorbance of this solution was measured, at the maximum at 257 nm using 0.1 M sodium hydroxide in the reference cell. The total content of paracetamol (C8H9NO2) in the medium was calculated taking 715 as the value of *A* (1%, 1 cm) at the maximum at 257 nm. The percentage concentration of paracetamol tablet was then calculated based on the sample absorbance and reference absorbance.

### 2.12. Statistical Analysis

Data was entered in Microsoft Excel 2010 and exported to Statistical Package for Social Science (IBM SPSS Statistics for Windows, Version 22.0) for analysis. Descriptive analysis was presented using frequency and percentage. The association between choosing local paracetamol and variables (age, sex, and educational background; the belief of expensive medicine is better in quality; and consumer's definition of drug quality) was carried out using bivariate and the multivariate logistic regression. Odds ratio, crude and adjusted, with 95% confidence interval, was reported in the analysis. The analyses were considered statistically significant when the *p* value was less than 0.05.

## 3. Result

Of the total 420 respondents, 403 responses were received, with a response rate of 95.95%. Majority of the respondents were females (61.8%), and the age range was from 18 to 82 years. The background characteristics are depicted in Table 1.

The majority of the respondents used locally manufactured paracetamol tablets for headache (81.6%), while 24% and 17% used it for fever and back pain, respectively, whereas 15% of the respondents used it for migraine and 15% for arthritis as it can be seen in Table 2.

Almost half of the respondents (49.1%) chose the local paracetamol over imported ones. The main reason of choosing local paracetamol was because of their good experience (62.1%) (Table 3). And the main reason for not choosing the local paracetamol was that the imported paracetamol was believed to confer higher quality (32.5%).

Moreover, 45.5% of the respondents had an experience of using imported paracetamol. Extra Panadol (60.9%), Amol (4.4%), and Tylenol (10.9%) were the most used imported paracetamol. Of the respondents, 27% believe expensive medicines are better in quality, and 68.5% did not believe when compared to the cheaper ones. About three-fourths (78.7%) of the participants believed that medicine manufactured abroad are higher quality when compared to the local ones. Moreover, 19.4% of the respondents believed that local paracetamol is lower in quality, 13.6% believed they are the of same quality, and 7.9% believed local paracetamol is better in quality than their imported counterparts. About three-fourths (76.9%) of the respondents believed that paracetamol having a quick relieve of pain is considered an effective medicine as it is shown in Table 4.

Bivariate and multivariate analysis was done to see if there is any association between choosing local paracetamol and independent variables such as age, sex, and educational background. It was found that age and sex did not show a statistically significant association. At the multivariate level, educational backgrounds such as elementary (AOR = 4.19, 95% CI: 1.251, 14.035) and junior (AOR = 2.4, 95% CI: 1.146, 5.028) were associated with choosing local paracetamol over the imported one. No formal education like secondary and tertiary level education were associated with choosing local paracetamol. In addition, in both bivariate and the multivariate levels, there was no statistically significant association between choosing local paracetamol and the dependent variables such as the belief of expensive medicines are better in quality and consumer's definition of drug quality.

### 3.1. Results of In Vitro Quality Assessment of the Locally Manufactured Paracetamol Tablet

#### 3.1.1. Weight Variation Test

**Figure 1:**
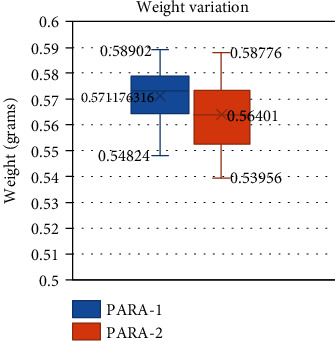
Weight variation test. PARA-1 (left) and PARA-2 (right).

The weight variation test of the two batches of local paracetamol, coded as PARA-1 and PARA-2, can be shown in Figure 1.

In the identification test, the resultant solution for the two batches was gradually appearing violent color which does not changed to red which indicates the presence of paracetamol active ingredient. The pharmacopeial assay test was done for both batches, PARA-1 had an actual absorbance of 0.5606, and the content of paracetamol was found to be 104.53%, complying with the British Pharmacopeial specification of 95-105% drug content. PARA-2 showed an actual absorbance of 0.53094, and the drug content was 99.001% which is in the range of the British pharmacopoeia [17, 18].

#### 3.1.2. Friability Test

**Figure 2:**
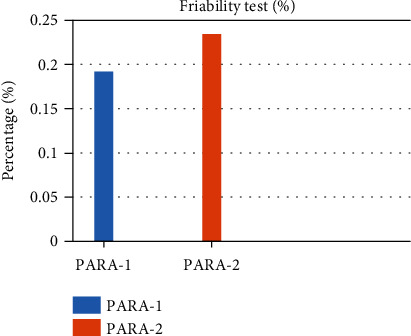
Friability test in percentage. PARA-1 (left) and PARA-2 (right).

The percentage friability of the two batches was within the pharmacopeial range as it can be depicted in Figure 2.

#### 3.1.3. Disintegration Test

The six tablets of PARA-1 used for the disintegration test of local paracetamol tablets took a range of 1.73 to 2.08 minutes to disintegrate completely.

In the second batch of local paracetamol tablets, PARA-2, the time taken for the six tablets to disintegrate completely was in the range of 1.15 to 1.30 minutes.

#### 3.1.4. Dissolution Test

The result of the dissolution test of the local paracetamol, with calculated the reference absorbance 0.396825, can be seen clearly in Table 5.

## 4. Discussion

This study tried to explore consumers' perception of generic medicines and in vitro quality assessment of locally manufactured paracetamol as it might shed light on the overall perception of generic medicine and branded medicines. Generally, about half (49.1%) of the respondents choose locally manufactured paracetamol over the imported ones. In similar studies, 51% of the participates opted for generic over-the-counter (OTC) analgesic medicines [19], and 75% preferred to have locally manufactured generic ones [8]. And Williams et al. found that 37.6% of the consumers agreed to take GM when the respondents were asked if they would rather take generic medicine rather than branded medicines [20], but two Indian studies showed that 25.6% [21] and 64% [2] of the study participants preferred using a brand formulation of paracetamol over its generic counterpart.

In our study, 78.7% of the consumers believed that medicine manufactured in abroad is of higher quality, and 68.5% did not believe expensive medicines are better in quality. In a relative study, 51% of the consumers agreed that the brand paracetamol has a higher quality compared to its generic counterparts [2]. However, a study performed in Malaysia revealed that 75% of the respondents disagreed that GMs were having a lower quality [22]. Still, 91% [19], 53.5% [22], and 57.6% of the participants believed that efficacy of GMs and branded medicines is the same, and 44% believed generic medicines are of poor quality [22].

ln the present study, respondents did not prefer local paracetamol to branded tablets due to its lower cost (99%). But in other comparative study, 69.4% [23] of the participants choose the cheaper generic over brand OTC medicines. In Saudi Arabia, 51.8% of the participants did not prefer using GMs because of their low price [24]. However, in similar studies, respondents preferred to have the cheapest medicine available [8, 25]. Besides, in another study, the majority of respondents associated low cost or no cost with lower quality of medicine [26]. The reasons why consumers did not opt for the local one maybe because they perceive cheaper medicines are inferior in quality [13, 23, 27, 28].

As per the findings of this study, 76.9% and 10.4% of the respondents considered paracetamol tablet conferring quick relief of pain and fewer side effects as an effective medicine, respectively. In a qualitative study done in South Africa, the participants considered that effective medicines are those which can alleviate symptoms without or little side effects [26]. Likewise, in other studies, it was shown that participants believe GMs have more side effects that branded medicines [20, 24]. Thus, the consumer's positive perception of GM mainly depends on its price advantage, access, and their experience. On the other hand, their negative perception depends on their lack of knowledge as they might perceive branded medicines are superior in quality due to the fact that some branded paracetamols are formulated as immediate release tablets [15].

### 4.1. In Vitro Analysis of Paracetamol Tablet

The two batches of paracetamol that were tested passed the test for identity, pharmacopeial assay, uniformity of weight, friability, disintegration, and dissolution. As per the result, the locally manufactured paracetamol did not appear to be lower in quality.

## 5. Conclusion

This study showed the consumers' general view and the reality of locally manufactured paracetamol tablet effectiveness. As the finding of the current study, consumers hold a negative view of the local paracetamol and tend to use the expensive brand paracetamol despite the fact that the laboratory specifications of the paracetamol tablet have met the standard. This study will be a benchmark for future studies, and a further nationwide research should be done to assess the knowledge and attitude of consumers and health care professionals on generic medicine to unveil the consumers practice and health care professional's attitude.

## 6. Limitation

Our study was focused on paracetamol tablets in a hope to get the consumers' general view of the GMs. Because only a few branded medicines are common among the consumers, we used the commonly known paracetamol tablet. We believe by specifying the study of a well-known medicine, it would give us the baseline data for future studies of the perception on generic medicines.

## Figures and Tables

**Table 1:** Sociodemographic characteristics of the participants (*N* = 403).

Variables		Frequency (*n*)	Percent (%)
Sex			
	Male	154	38.2
	Female	249	61.8
Age			
	18-24	71	17.6
	25-34	115	28.5
	35-44	100	24.8
	45-54	62	15.4
	55-64	36	8.9
	65 and above	19	4.7
Marital status			
	Married	274	68.0
	Single	113	28.0
	Divorced	9	2.2
	Widowed	7	1.7
Educational level			
	No formal education	11	2.7
	Elementary	22	5.5
	Junior	64	15.9
	Secondary	183	45.4
	Tertiary level	123	30.5

**Table 2:** Conditions of using local paracetamol.

Conditions	Frequency (*n*)	Percent (%)
Headache	329	81.6
Relieve pain	91	22.6
Migraine headache	61	15.1
Arthritis	61	15.1
Backache	69	17.1

**Table 3:** Reasons for choosing or not choosing local paracetamol.

Variables			Frequency (*n*)	Percent (%)
Choosing of paracetamol as a pain killer	Local		198	49.1
	Imported		197	48.9
	Both		8	2
Reasons for choosing local paracetamol	Same as imported	Yes	7	3.5
		No	191	96.5
	It is cheaper	Yes	2	1
		No	196	99
	Used before and worked well	Yes	123	62.1
		No	75	37.9
	Availability	Yes	26	6.5
		No	172	42.7
Reasons for not choosing local paracetamol	Used before and did not worked well	Yes	35	8.7
		No	162	82.2
	Cheaper so inferior quality	Yes	3	7
		No	194	48.1
	Took longer to have an effect	Yes	40	9.9
		No	157	39
	Imported one have higher quality	Yes	131	32.5
		No	66	16.4
	Other people said its ineffective	Yes	7	1.7
		No	190	47.1

**Table 4:** Consumers' definition of drug quality.

Quality drug		Frequency (*n*)	Percentage (%)
Quick relieve of pain	Yes	310	76.9
	No	93	23.1
Fewer side effect	Yes	118	10.4
	No	285	89.6
Higher cost	Yes	7	1.7
	No	396	98.6

**Table 5:** Dissolution test of the two batches of local paracetamol.

Dissolution test of PARA 1		
Sample No.	UV reading	Percentage (%)
Sample1	0.4115	103.70%
Sample 2	0.3987	100.40%
Sample 3	0.4413	111.20%
Sample 4	0.402	101.30%
Sample 5	0.4004	100.90%
Sample 6	0.4759	119.90%
Average	0.42163	106.25%
Dissolution test of PARA 2		
Sample No.	UV reading	Percentage (%)
Sample1	0.40155	101.19%
Sample 2	0.4186	105.50%
Sample 3	0.4355	109.75%
Sample 4	0.4095	103.20%
Sample 5	0.4419	111.36%
Sample 6	0.4304	108.47%
Average	0.4229	1.06571%

## Data Availability

The complete data set used and/or analyzed during the current study is available from the corresponding author and can be accessed upon reasonable request.
