# Corrigendum to “Consumers' Perception of Generic Medicines and Evaluation of In Vitro Quality Control Parameters of Locally Manufactured Paracetamol Tablets in Asmara, Eritrea: A Cross-Sectional Study”

**DOI:** 10.1155/2021/9808590

**Published:** 2021-06-11

**Authors:** Idris Mohammed Idris, Diyae Nesredin Hassan, Hanan Abdelkadir Hassen, Rahwa Zerabruk Araya, Dawit G. Weldemariam

**Affiliations:** ^1^Department of Anesthesia, Eritrean Air Force Military Hospital, Asmara, Eritrea; ^2^Department of Medical Sciences, Orotta College of Medicine and Health Sciences, Asmara, Eritrea; ^3^Department of Pharmacy, Hazhaz Zonal Referral Hospital, Asmara, Eritrea

In the article titled “Consumers' Perception of Generic Medicines and Evaluation of In Vitro Quality Control Parameters of Locally Manufactured Paracetamol Tablets in Asmara, Eritrea: A Cross-Sectional Study” [1], there were errors in Tables 3 and 5, as follows:

- In Table 3, the values included in the percentage column are incorrect due to an error introduced when transferring data from a spreadsheet to statistics software package
- In Table 5, there was a typographical error in the last cell in the last row of the percentage column where 1.0657% should be corrected to 106.57%

The authors confirm that this does not affect the results and conclusions of the article, and the corrected tables are shown below.

## Figures and Tables

**Table 3:** Reasons for choosing or not choosing local paracetamol.

Variables			Frequency (*n*)	Percent (%)
Choosing of paracetamol as a pain killer	Local		198	49.1
	Imported		197	48.9
	Both		8	2

Reasons for choosing local paracetamol	Same as imported	Yes	7	3.5
		No	191	96.5
	It is cheaper	Yes	2	1
		No	196	99
	Used before and worked well	Yes	123	62.1
		No	75	37.9
	Availability	Yes	26	13.1
		No	172	86.9

Reasons for not choosing local paracetamol	Used before and did not worked well	Yes	35	17.8
		No	162	82.2
	Cheaper so inferior quality	Yes	3	1.5
		No	194	98.5
	Took longer to have an effect	Yes	40	20.3
		No	157	79.9
	Imported one have higher quality	Yes	131	66.5
		No	66	33.5
	Other people said its ineffective	Yes	7	3.6
		No	190	96.4

**Table 5:** Dissolution test of the two batches of local paracetamol.

Dissolution test of PARA 1		
Sample no.	UV reading	Percentage (%)
Sample 1	0.4115	103.70%
Sample 2	0.3987	100.40%
Sample 3	0.4413	111.20%
Sample 4	0.402	101.30%
Sample 5	0.4004	100.90%
Sample 6	0.4759	119.90%
Average	0.42163	106.25%
Dissolution test of PARA 2		
Sample no.	UV reading	Percentage (%)
Sample 1	0.40155	101.19%
Sample 2	0.4186	105.50%
Sample 3	0.4355	109.75%
Sample 4	0.4095	103.20%
Sample 5	0.4419	111.36%
Sample 6	0.4304	108.47%
Average	0.4229	106.57%

## References

[1] Idris I. M., Hassan D. N., Hassen H. A., Araya R. Z., Weldemariam D. G. (2021). Consumers’ Perception of Generic Medicines and Evaluation of In Vitro Quality Control Parameters of Locally Manufactured Paracetamol Tablets in Asmara, Eritrea: A Cross-Sectional Study. *BioMed Research International*.

